# Prevalence of the American Heart Association's “Ideal Cardiovascular Health” Metrics in a Rural, Cross‐sectional, Community‐Based Study: The Heart of New Ulm Project

**DOI:** 10.1161/JAHA.113.000058

**Published:** 2013-06-21

**Authors:** Jacqueline I. Kim, Arthur Sillah, Jackie L. Boucher, Abbey C. Sidebottom, Thomas Knickelbine

**Affiliations:** 1 Minneapolis Heart Institute Foundation, Minneapolis, MN (J.I.K., J.L.B.); 2 Allina Health, Minneapolis, MN (A.S., A.C.S.); 3 Minneapolis Heart Institute, Minneapolis, MN (T.K.)

**Keywords:** cardiovascular diseases, coronary disease, prevention, risk factors

## Abstract

**Background:**

The American Heart Association (AHA) recently created the construct of “ideal cardiovascular health” based on 7 cardiovascular health metrics to measure progress toward their 2020 Impact Goal. The present study applied this construct to assess the baseline cardiovascular health of a rural population targeted with a community‐based cardiovascular disease prevention program.

**Methods and Results:**

The sample consists of 4754 New Ulm, Minn, adult residents who participated in either the 2009 or 2011 community heart health screenings offered by the Hearts Beat Back: The Heart of New Ulm (HONU) Project (mean age 52.1 years, 58.3% women). Data collected at the screenings were analyzed to replicate the AHA's ideal cardiovascular health measure and the 7 metrics that comprise the construct. Screening participants met, on average (±SD), 3.4 (±1.4) ideal cardiovascular health metrics. Only 1.0% of participants met the AHA's definition of ideal health in all metrics and 7.1% met ≤1 ideal health metric. Higher proportions of women met the ideal category in all metrics except physical activity. Women over the age of 60 were less likely to meet the ideal category for cholesterol and hypertension than were men in the same age range.

**Conclusion:**

Prevalence of ideal cardiovascular health is extremely low in this rural population. To make progress toward the 2020 Impact Goal, targeted community‐based interventions must be implemented based on the most prevalent cardiovascular risk factors.

## Introduction

Cardiovascular disease (CVD) is the leading cause of morbidity and mortality in the United States, causing 1 of every 3 deaths each year. It is an economically burdensome disease, with CVD‐related costs comprising 17% of the nation's total health expenditures.^1^ Coronary heart disease (CHD), in particular, makes up the majority of cardiovascular events for men and women, causing 1 of every 6 deaths in the United States and an average of 16.6 life‐years lost due to myocardial infarctions.^2^

Trends indicate that despite an ≈50% decrease in the age‐adjusted mortality rate due to coronary disease since 1980 and significant reductions in major CVD risk factors such as smoking, elevated cholesterol, and high blood pressure (BP), CVD‐related events and the prevalence of obesity and diabetes continue to rise in the United States.^2–4^ The American Heart Association's (AHA) 2010 Impact Goal focused on the reduction of CHD and stroke, as well as their associated risk factors. To address the increasing need for prevention as well as treatment of CVD, the AHA broadened the scope of the 2020 Impact Goals to include not only reducing deaths from all CVD and stroke but also improving the cardiovascular health of the population as a whole. To measure progress toward these prevention‐related goals, the AHA defined the construct of “ideal cardiovascular health” based on 7 metrics: smoking status, body mass index (BMI), nutritional intake, physical activity level, and levels of BP, blood glucose, and total cholesterol.^3^

The 7 metrics selected by the AHA to evaluate cardiovascular health have been associated with lower risk of CHD and CVD, as well as lower mortality rates from CVD and all causes, and greater longevity in both men and women.^5–7^ Previous studies have determined the prevalence of AHA‐defined ideal cardiovascular health in the US population via analysis of National Health and Nutrition Examination Survey (NHANES) data as well as in a middle‐aged community‐based population.^8–11^ However, the AHA's metrics have not been broadly studied or previously applied to a rural population. The purpose of this study was to assess the baseline cardiovascular health of the rural New Ulm, Minn, adult population being targeted by the Hearts Beat Back: The Heart of New Ulm (HONU) Project CVD prevention program using the AHA's cardiovascular health metrics to add to the literature about the application of the AHA measures to a rural population.

## Methods

### Design and Setting

This study used a cross‐sectional examination of baseline data collected in the 2009 and 2011 community cardiovascular risk screenings for the HONU Project.^12–13^ HONU was initiated in 2009 as a 10‐year community‐based cardiovascular health intervention designed to reduce the number of myocardial infarctions and CVD risk factors among residents living in the 56073 zip code, which encompasses the city of New Ulm, Minn. The project is a collaborative partnership of Allina Health, the Minneapolis Heart Institute Foundation, the New Ulm Medical Center, and the community of New Ulm. The target zip code has an adult population of 13 290 according to 2010 census data and is located in Brown County, which is 97.4% white and in an agricultural region of the state. Community‐wide cardiovascular risk screenings, a primary component of the intervention, were offered in 2009 and 2011 during 2 screening periods from April to December 2009 and May to December 2011. HONU recruited participants at a total of 175 screening events (109 in 2009 and 66 in 2011) held at local worksites and central gathering places in the city, including recreation centers, religious institutions, and the New Ulm Medical Center.^13^ While HONU targets 40‐ to 79‐year‐old residents, given that this is the demographic within which near‐term preventable myocardial infarctions are most frequently observed, the screenings were open to all adults (defined as 18 years or older). Use of the HONU screening data was approved by the Allina Institutional Review Board and all participants gave informed consent.

### Study Sample

A total of 4166 adults from the target zip code attended screenings in 2009, and 2521 residents attended in 2011 (742 came for the first time in 2011). For those individuals who attended both screenings (1779), we included data only from the first screening because these values offered a baseline assessment. There were 4908 residents who participated in either 2009 or 2011. Of these, 4754 are included in the final sample because they have complete data on the 7 measures needed for the analysis.

### Data Collection

Screening evaluations in both years were conducted by trained health professionals from New Ulm Medical Center. There were no meaningful changes in the data collection protocol or in the elements collected between the 2009 and 2011 screenings. The assessment included collection of the following: demographics, insurance status, personal and family health history, smoking status, physical activity, fruit and vegetable consumption, psychosocial characteristics, anthropometric and physiological measurements (height, weight, waist circumference, and BP), and cardiovascular health‐related biometric data (total cholesterol, low‐density lipoprotein [LDL] cholesterol, high‐density lipoprotein [HDL] cholesterol, triglycerides, high‐sensitivity C‐reactive protein, glucose, and glomerular filtration rate). Detailed descriptions of the HONU Project and the data collected from the community cardiovascular risk screenings have been described previously.^12–13^

### Definition of Cardiovascular Health Metrics

The AHA's definition of ideal cardiovascular health is identified as the simultaneous presence of 7 favorable cardiovascular health characteristics: nonsmoking, BMI <25 kg/m^2^, ≥150 min/wk of moderate intensity exercise or the vigorous intensity exercise equivalent, consumption of a diet consistent with a Dietary Approaches to Stop Hypertension (DASH)‐type dietary pattern, untreated total cholesterol <200 mg/dL, untreated BP <120/<80 mm Hg, and untreated fasting glucose <100 mg/dL.^3^ Table 1 shows how we used HONU screening data to duplicate these measures where possible and where our measures deviated.

**Table 1.** Definition of Cardiovascular Health Metrics

Measure	Measurement for Current Study	Categories
Smoking status*	Screening participants self‐identified as “never,” “former,” or “current” smokers.	Ideal: self‐reported “never” or “former” smoker Poor: self‐reported “current” smoker
BMI	Height and weight without shoes collected by medical staff at screenings. Measurements provided in kg/m^2^.	Ideal: <25 kg/m^2^ Intermediate: 25 to 29 kg/m^2^ Poor: ≥30 kg/m^2^
Physical activity	Participants self‐reported weekly levels of moderate and vigorous intensity physical activity using the BRFSS items.^9^ Vigorous activity minutes doubled to provide an estimate of moderate intensity physical activity and added to self‐reported moderate activity minutes to create a measure of total physical activity at a moderate intensity.	Ideal: 150+ min/wk Intermediate: 1 to 149 min/wk Poor: 0 min/wk
Diet*	HONU used a single item measuring daily servings of fruit and vegetable consumed.	Ideal: ≥5 servings/d Intermediate: 3 to 4 servings/d Poor: ≤2 servings/d
Total serum cholesterol	Total cholesterol measured from a fasting venous blood draw analyzed at Allina Medical Laboratories of Allina Health. Results were categorized into AHA levels.	Ideal: <200 mg/dL, untreated Intermediate: 200 to 239 mg/dL or treated to goal levels Poor: ≥240 mg/dL, regardless of treatment
BP	BP measured twice after 3 minutes of rest in a seated position by trained medical staff using the Sun Tech 247, an automated blood pressure measuring device, with a properly sized cuff. Average of the 2 readings used to categorize results into AHA levels.	Ideal: <120/80 mm Hg, untreated Intermediate: 120 to 139/80 to 88 mm Hg or treated to goal levels Poor: ≥140/90 mm Hg
Fasting blood glucose	Fasting blood glucose measured from a fasting venous blood draw using standard analytic technique and categorized into AHA levels.	Ideal: <100 mg/dL, untreated Intermediate: 100 to 125 mg/dL or treated to goal levels Poor: ≥126 mg/dL

BMI indicates body mass index; AHA, American Heart Association; HONU, Heart of New Ulm; BRFSS, Behavioral Risk Factor Surveillance System; DASH, dietary approaches to stop hypertension; BP, blood pressure.

* The AHA categorized individuals' health as ideal if they had never smoked or quit >12 months earlier, intermediate if they had quit ≤12 months earlier, and poor if they currently smoked. The HONU screen did not ask former smokers about time elapsed since quitting. Because most former smokers will have quit >1 year earlier, all were grouped into the ideal category.

* The AHA categories included the 5 nutrition components of the DASH Diet. The HONU survey did not include 4 of these 5 items: consumption of fish, fiber‐rich whole grains, sodium, and sugar‐sweetened beverages.

### Cardiovascular Health Metrics Score

A cardiovascular health metrics score was calculated for each screening participant by recoding each of the cardiovascular health metrics as dichotomous variables in which 1 point was assigned for ideal status and 0 points for intermediate or poor status. The sum of these indicates how many individual measures a participant met at the ideal level.

### Statistical Analyses

All analytical procedures were conducted using SPSS statistical packages version 18 (SPSS Inc), with statistical significance considered at 2‐sided α value of .05. Age‐ and sex‐specific estimates are also presented. Means and SDs for continuous variables (all continuous measures were normally distributed) and percentages for categorical measures were calculated. Pearson χ^2^ tests were used for comparisons of age and sex differences for all tables, and an unpaired *t* test was used to compare the mean number of ideal metrics (see Table 4).

## Results

### Demographic Characteristics

The mean (±SD) age of the screening participants was 52.1 (±16) years (Table 2). Participation among women was higher than that of men (58.3% versus 41.7%) and the vast majority self‐reported their race as white (95.7%). Most participants had some level of postsecondary education (66.9%). Almost all screening participants had health insurance (96.7%). A comparison of screening participants to 2010 US Census figures for the zip code showed slightly higher female participation in the screenings (7% points) but similar proportions of residents over the age of 60. A higher proportion of residents aged 40 to 59 were represented in the screening data compared with the distribution in the Census data, and those aged 18 to 39 were less represented in the screening data. Given that the HONU program targets those aged 40 to 79, this overrepresentation of the 40‐to‐59 age group is not surprising.

**Table 2.** Characteristics of HONU Screening Participants in 2009 or 2011 (N=4754)

Characteristics	HONU Data, n (%)	2010 Census Data, n (%)
Age, mean y (SD)	52.1 (16.0)	
18 to 39	1017 (21.4)	4373 (32.9)
40 to 59	2234 (47.0)	4885 (36.8)
60+	1503 (31.6)	4032 (30.4)
Sex, No. (%)		
Male	1981 (41.7)	6494 (48.9)
Female	2773 (58.3)	6796 (51.1)
Race, No. (%)		
White	4548 (95.7)	—
Not white	197 (4.1)	—
Missing	9 (0.2)	—
Education, No. (%)		
Less than high school	191 (4.0)	—
High school or GED	1340 (28.2)	—
Tech or associate or some college	1693 (35.6)	—
College degree	1076 (22.6)	—
Graduate or professional degree	412 (8.7)	—
Missing	42 (0.9)	—
Health insurance, No. (%)		
Covered	4597 (96.7)	—
Not covered	128 (2.7)	—
Missing	29 (0.6)	—
Self‐reported disease history, No. (%)		
Heart disease	332 (7.0)	—
Diabetes (not gestational)	321 (6.8)	—

If a person participated in a screening in both 2009 and 2011, data presented are from their first screening. HONU indicates Heart of New Ulm; GED, general educational development.

### Clinical Characteristics

Ideal status was reached by the majority of screening participants for 3 cardiovascular health metrics: smoking status, physical activity levels, and fasting blood glucose (Table 3). However, the majority were categorized as being of poor or intermediate health in the remaining 4 measures of BMI, fruit and vegetable consumption, total serum cholesterol levels, and BP. Levels of self‐reported diabetes were low (6.8%) despite a substantial proportion of the study population (30.5%) having elevated blood glucose levels indicative of prediabetes or diabetes.

**Table 3.** Prevalence of Cardiovascular Health Metrics Among 2009 and 2011 Heart of New Ulm Screening Participants (N=4754)

Cardiovascular Health Metrics	All Ages				Age Groups				18 to 39 y			40 to 59 y			60+ y		
	Total (N=4754), %	Women (n=2773), %	Men (n=1981), %	*P* Value	18 to 39 y (n=1017), %	40 to 59 y (n=2234), %	60+ y (n=1503), %	*P* Value	Women (n=601), %	Men (n=416), %	*P* Value	Women (n=1297), %	Men (n=937), %	*P* Value	Women (n=875), %	Men(n=628), %	*P* Value
Smoking status																	
Ideal (never/former)	90.0	90.9	88.6	0.009	84.4	88.8	95.6	<0.001	85.9	82.2	0.116	89.7	87.5	0.111	96.3	94.6	0.101
Poor (current)	10.0	9.1	11.4		15.6	11.2	4.4		14.1	17.8		10.3	12.5		3.7	5.4	
Body mass index (BMI), kg/m^2^																	
Ideal (healthy weight, BMI <25)	26.8	33.5	17.4	<0.001	38.7	24.9	21.4	<0.001	44.1	31.0	<0.001	34.0	12.4	<0.001	25.4	15.8	<0.001
Intermediate (overweight, BMI 25 to 29)	34.8	30.4	41.0		31.8	34.3	37.6		27.0	38.7		29.3	41.3		34.4	42.0	
Poor (obese, BMI ≥30)	38.4	36.1	41.6		29.5	40.7	41.1		29.0	30.3		36.7	46.3		40.2	42.2	
Physical activity																	
Ideal (≥150 min/wk)	63.7	64.2	63.1	0.750	66.6	66.0	58.4	<0.001	71.4	59.6	<0.001	65.3	67.0	0.688	57.5	59.7	0.675
Intermediate (1 to 149 min/wk)	34.0	33.6	34.6		32.5	32.3	37.6		27.6	39.7		33.3	31.3		38.5	36.3	
Poor (0 min/wk)	2.3	2.3	2.2		0.9	1.7	4.0		1.0	0.7		1.7	1.7		4.0	4.0	
Fruit and vegetables																	
Ideal (sufficient ≥5 servings/d)	16.4	19.9	11.4	<0.001	13.6	15.0	20.2	<0.001	14.6	12.0	<0.001	19.0	9.5	<0.001	24.8	13.9	<0.001
Intermediate (insufficient 3 to 4 servings/d)	36.9	43.6	27.7		32.4	35.9	41.5		39.4	22.1		42.7	26.6		47.7	33.0	
Poor (insufficient 0 to 2 servings/d)	46.7	36.5	60.9		54.1	49.0	38.3		45.9	65.9		38.2	63.9		27.5	53.2	
Total serum cholesterol, mg/dL																	
Ideal (normal and untreated, <200)	41.8	42.8	40.4	<0.001	71.9	39.6	24.8	<0.001	76.0	65.9	0.002	41.6	36.7	<0.001	21.8	29.0	<0.001
Intermediate (200 to 239 or treated to normal)	45.8	42.4	50.5		22.6	45.8	61.3		19.0	27.9		42.2	50.9		58.7	65.0	
Poor (≥240)	12.4	14.8	9.1		5.5	14.6	13.8		5.0	6.3		16.2	12.4		19.4	6.1	
Blood pressure, mm Hg																	
Ideal (normal and untreated, <120/80)	30.9	36.7	22.9	<0.001	50	21.1	20.0	<0.001	60.9	34.1	<0.001	39.4	20.3	<0.001	16.0	19.3	<0.001
Intermediate (120 to 139/80 to 89 or treated to normal)	51.1	48.2	55.2		42.0	47.5	62.7		33.4	56		48.4	60.0		69.7	66.1	
Poor (≥140/90)	18.0	15.1	21.9		8.1	31.4	17.4		5.7	9.9		12.2	19.7		14.3	14.6	
Fasting blood glucose, mg/dL																	
Ideal (<100, untreated/no history of diabetes)	69.5	74.4	62.5	<0.001	90.3	70.1	54.4	<0.001	91.8	88.0	0.046	75.9	62.2	<0.001	60.2	46.2	0.001
Intermediate (100 to 125 or treated to normal)	24.4	20.1	30.4		8.1	24.8	34.9		6.3	10.6		19.4	32.2		30.6	40.8	
Poor (≥126)	6.1	5.5	7.1		1.7	5.1	10.8		1.8	1.4		4.7	5.5		9.1	13.1	

The mean (±SD) number of health measures individuals achieved ideal status was 3.4 (±1.4) (Table 4). Only 49 (1.0%) individuals were identified as having ideal cardiovascular health by meeting ideal health status in all 7 measures. Fewer than half (44.5%) of the total sample met ≥4 ideal metrics.

**Table 4.** Number of Cardiovascular Health Metrics Met Among 2009 and 2011 Heart of New Ulm Screening Participants (N=4754)

	All Ages				Age Groups				18 to 39 y			40 to 59 y			60+ y Age Group		
	Total (N=4754)	Women (n=2773)	Men (n=1981)	*P* Value	18 to 39 y (n=1017)	40 to 59 y (n=2234)	60+ y (n=1503)	*P* Value	Women (n=601)	Men (n=416)	*P* Value	Women (n=1297)	Men (n=937)	*P* Value	Women (n=875)	Men (n=628)	*P* Value
Number of ideal metrics, mean (SD)	3.4 (1.4)	3.6 (1.4)	3.1 (1.2)	<0.001	4.2 (1.3)	3.4 (1.4)	2.9 (1.2)	<0.001	4.5 (1.3)	3.7 (1.2)	<0.001	3.6 (1.5)	3.0 (1.2)	<0.001	3.0 (1.2)	2.8 (1.1)	<0.001
Number of ideal metrics, %																	
0	0.6	0.5	0.7	<0.001	0.2	0.9	0.5	<0.001	0.0	0.5	<0.001	0.9	0.7	<0.001	0.2	0.8	<0.001
1	6.5	5.5	7.9		2.2	6.6	9.3		1.8	2.6		4.6	9.3		9.4	9.2	
2	21.1	18.1	25.4		8.3	21.2	29.7		5.5	12.3		18.0	25.7		27.0	33.6	
3	27.3	24.1	31.8		19.7	28.1	31.2		15.5	24.7		23.7	34.0		30.4	32.3	
4	22.6	23.3	21.6		28	22.5	19.2		25.1	32.2		24.1	20.2		20.9	16.7	
5	14.4	17.5	10.1		26.5	13.3	7.7		30.6	20.7		17.0	8.2		9.0	5.9	
6	6.5	9.3	2.5		13.2	6.3	2.3		18.3	5.8		9.6	1.7		2.9	1.4	
7	1.0	1.7	0.1		2.0	1.2	0.1		3.2	0.2		2.0	0.1		0.2	0.0	
Categories for extreme levels, %																	
0 to 1	7.1	6.0	8.6	<0.001	2.4	7.4	9.8	<0.001	1.8	3.1	0.181	5.6	10.0	<0.001	9.6	10.0	0.781
≥6	7.5	11.0	2.6	<0.001	15.1	7.5	2.4	<0.001	21.5	6.0	<0.001	11.6	1.8	<0.001	3.1	1.4	0.0333
Summary categories, %																	
0 to 3	55.5	48.2	65.7	<0.001	30.3	56.7	70.7	<0.001	22.8	41.1	<0.001	47.3	69.8	<0.001	67.0	76.0	<0.001
4 to 7	44.5	51.8	34.3		69.7	43.3	29.3		77.2	58.9		52.7	30.2		33.0	24.0	

Comparisons between age categories (Table 3) indicated higher proportions meeting ideal status for smoking as well as fruit and vegetable intake as age increased. For physical activity, the proportion of individuals meeting ideal levels was the same for those aged 18 to 39 and 40 to 59, with a decrease in the 60+ age group. For BMI, total cholesterol, glucose, and BP, those in the younger age groups were more likely to meet ideal status than those in the older age groups.

When sex differences were examined within age categories (Table 3), no differences were seen in smoking. Women aged 18 to 39 were more likely to be physically active than were men, but no sex differences were seen in the older age groups. For BMI, a higher proportion of women met the ideal category in all age groups, but the trend was not linear, with the widest sex gap among those aged 40 to 59. Both cholesterol and hypertension showed higher proportions of women aged 18 to 39 and 40 to 59 meeting the ideal category, but the opposite was true for those over 60. The metrics also showed large sex differences in BP, with a 26.8% point difference for individuals aged 18 to 39 and a 19.1% point difference among those aged 40 to 59. For the metrics of fruit and vegetable consumption and glucose, differences between men and women increased with age. For fruit and vegetable consumption, the younger age group showed only a 2.6% point difference, which increased to differences of 9.5 among those aged 40 to 59 and 10.9 among those over age 60. Glucose showed a similar pattern with a sex difference of 3.8 between the youngest age group, 13.7 among 40‐ to 59‐year‐olds, and 14 in the oldest group.

## Discussion

In this cross‐sectional study using data from community cardiovascular risk screenings conducted in the rural Minnesotan community of New Ulm, we found that achievement of the AHA's definition of ideal cardiovascular health was extremely low. Particular areas of concern for the New Ulm population were BMI and fruit and vegetable intake, with the majority of the population falling into the poor health categories for these measures. Women made up a higher proportion of the ideal category than men in BP and cholesterol in every age category except for those aged 60+, a finding that is consistent with expected age‐/sex‐specific trends for postmenopausal women.^14–15^ The identification of substantial numbers of participants with glucose levels indicating prediabetes and diabetes despite low levels of self‐reported diabetes is consistent with Centers for Disease Control and Prevention estimates of undiagnosed prediabetes and diabetes within the US population. Diabetes is estimated to affect 25.8 million Americans, of whom 7.0 million are undiagnosed and an additional 79 million are estimated to have prediabetes—the majority of whom are unaware of their elevated glucose levels.^16^

Compared with the rest of the state of Minnesota, HONU screening participants have higher levels of physical activity (63.7% versus 52.7%) and are nonsmokers (90.0% versus 83.2%) but have poorer fruit and vegetable intake (16.4% versus 21.9%) and higher BMIs (26.8% healthy weight and 38.4% obese versus 36.8% healthy weight and 25.4% obese).^17^ The fact that levels of physical activity within the study population were high compared with state and national averages may be explained in part by New Ulm's higher number of recreational facilities per 100 000 residents (20) than the state of Minnesota (12) or the national benchmark (16).^18^

As with state comparisons, when HONU results are compared with 2010 data from Hennepin County, an urban Minnesota county that encompasses Minneapolis, the largest city in Minnesota, the New Ulm residents tended to be worse in the categories of BMI and fruit and vegetable consumption. HONU participants were less likely to meet the healthy weight compared with Hennepin County residents (26.8% versus 46.9%) and more likely to be obese (38.4% versus 20.4%). A significantly smaller proportion of HONU participants consumed ideal amounts of fruits and vegetables (≥5 servings daily) than Hennepin County residents (16.4% versus 37.3%). Proportions of nonsmokers among the urban and rural population samples were comparable (90.0% HONU versus 88.0% Hennepin County); other measures were not available.^19^ Since passage of the Freedom to Breathe legislation in 2007 in which smoking was banned in most workplaces and public indoor spaces in Minnesota, smoking rates and nonsmokers' exposure to tobacco have been reduced, which may help account for high proportions of nonsmokers in the HONU and Minnesota samples.^20^

Our findings are similar to the results of 2 previously published studies that applied the AHA's ideal cardiovascular health metrics to a community‐based population and a nationally representative sample of the US population, both of which identified a low prevalence of ideal cardiovascular health.^10–11^ To the best of our knowledge, our study is the first to apply the metrics to a rural American community where median incomes are $13 000 less than the state average and higher education levels are 9% lower than the state average. Rural communities are often considered to have health inequities compared with metropolitan communities given access issues, such as decreased access to care and high‐quality food options. Given that ≈21% of the American population resides in a rural population, according to the most recent US Census data, the results of this study provide insight into public health considerations that have the potential to affect nearly a quarter of the American population.

Bambs et al^10^ applied the AHA's 7 cardiovascular health metrics to another community‐based cardiovascular health intervention program in Allegheny County, Pa, called the Heart Strategies Concentrating on Risk Evaluation (Heart SCORE) Study. The Heart SCORE analysis was similar to this analysis, but its participant sample differed in racial composition and geographic location. By design, the Heart SCORE study aimed to enroll only 2000 participants, with a majority of participants identified as intermediate to high Framingham risk, and 10% with established CVD. HONU's screenings were open to all adults in the community regardless of underlying health status and had no limit on the number of enrollees. The Heart SCORE study included both blacks and whites, whereas HONU screening participants were predominantly white, which is representative of the racial composition of Brown County. Also, Allegheny County is an urban population that includes the city of Pittsburgh, while New Ulm is a rural population.

Ideal cardiovascular health was much lower in the Heart SCORE population than in the HONU population, with only 1 of 1933 screening participants attaining ideal health in all 7 metrics. This difference could be accounted for by the lower mean age of the HONU sample than that of the Heart SCORE sample, given that cardiovascular risk factors are known to increase with age,^2,21^ or by the racial diversity of the Heart SCORE sample, as the burden of cardiovascular risk factors and disease disproportionately affects blacks in America.^2,22^ The findings of the Heart SCORE and HONU total cohorts were similar in that women comprised a larger proportion of those achieving the ideal health categories in the majority of the metrics (smoking status, BMI, BP, and fasting plasma glucose status). However, men outperformed women in attaining ideal health status in the areas of physical activity and total cholesterol levels in the Heart SCORE study, which was not true in the HONU study.

In comparisons between the white populations of each study, proportions meeting ideal levels in the metrics of BMI and fasting blood glucose were comparable. However, the New Ulm sample had significantly higher proportions meeting the definition of ideal health in the metrics of physical activity (63.7% versus 27.4%), total cholesterol levels (41.8% versus 19.6%), and BP (30.9% versus 18.9%). These differences could potentially be explained by differences in health insurance in the 2 communities. Although information on the number of individuals with health insurance was not available for the Allegheny County population, the New Ulm population sample had a remarkably high level of coverage (96.7%) and lack or loss of insurance is known to be related to declines in health and higher levels of unmet health needs and mortality.^23–25^ Another factor that might help to explain differences between the 2 populations is that of limited access to healthy foods. Only 1% of Brown County, which houses the town of New Ulm, both lives in poverty and does not live within 10 miles of a grocery store in a nonmetropolitan setting, whereas Allegheny County had a limited access to healthy foods ranking of 6%.^18^

Yang et al^11^ applied the AHA's cardiovascular health metrics to a nationally representative sample of American adults using data from the NHANES. Limiting data from the NHANES study to that collected from 2005 to 2010, the most recent body of data included in the study, we found that the New Ulm data showed similar trends to those of the NHANES data. Comparable proportions of both studies met ≤1 ideal health metrics (HONU 7.1%, NHANES 8.8%) and ≥6 ideal metrics (HONU 7.5%, NHANES 8.8%). Splitting the population samples into 2 groups above and below the median number of ideal cardiovascular metrics attained, a similar proportion of New Ulm residents who met ≥4 metrics versus the sample representative of the American population (44.5% versus 47.7%).

When the cardiovascular health metrics were analyzed separately, the proportion of New Ulm residents meeting ideal levels in physical activity was higher than that of the NHANES population (63.7% versus 45.2%) and a smaller proportion of the population was classified as meeting poor status in physical activity (2.3% versus 31.9%), although New Ulm had a slightly higher obesity rate (38.4% versus 34.1%) and a lower proportion of its population met ideal BP levels (30.9% versus 42.8%). The high rate of physical activity among New Ulm participants may again be explained in part by greater access to recreational facilities within the community.^18^

The results from the current study and the others using the AHA metrics show how these measures are useful for assessing risks at a population level to inform prevention efforts. In practice, results from the AHA measures could be used in communities to help assess baseline cardiovascular health and identify areas to focus interventions, especially those focused on policies, systems, and environmental changes. The information gained from this analysis corroborates the results of the 2009 HONU cardiovascular risk screenings, which identified weight and nutrition as high‐priority measures for health interventions in New Ulm. Specifically, high prevalence of obese and overweight individuals in the New Ulm population appeared to be correlated with poor nutrition, since ideal physical activity levels were high within the community. Based on the findings of the screenings, HONU program planners have directed resources toward improving the community's food environment by partnering with local food service providers (ie, restaurants, grocery stores, and farmers markets) to increase community access to and consumption of fruits and vegetables, in addition to decreasing portion sizes.^13^ Additional training has been provided to individuals interested in community‐supported agriculture to increase locally grown fruits and vegetables and improve community access to these higher‐quality food options.

Rural populations are unique compared with metropolitan areas in that there is often 1 health care system providing care to the population. Because there is only 1 health care provider in New Ulm, it is possible for the HONU Project to use the electronic health record (EHR) as the primary surveillance tool to study the health of the population. The AHA's cardiovascular health metrics, however, are not systematically captured through the EHR, and in the absence of the HONU Project these factors would not be easily measurable. For these metrics to be captured on an ongoing basis, systems would need to be developed to ask the lifestyle and behavior questions not addressed in the traditional health care visit, perhaps in the form of a health risk assessment questionnaire given during an annual physical examination.

In addition to the use of the AHA metrics for assessing population‐level risks, incorporating the measures into the EHR allows for more systematic assessment of the cardiovascular risk factors of individual patients and could serve as an enhanced tool for clinical practice to counsel patients about those risk factors. However, this potential benefit is challenged by barriers of time to collect and discuss information. Tai‐Seale et al^26^ found that primary care visits run a median length of 15.7 minutes, during which a median of 6 topics are discussed; ≈5 minutes are spent on the patient's chief complaint and all remaining topics receive 1.1 minutes. Realistically, a provider would be hard‐pressed to complete the AHA's cardiovascular health assessment and address the patient's cardiovascular risk factors within 1.1 minutes. In a 2006 case vignette survey of a random sample of 12 000 US family physicians and general internists, 53.4% of respondents identified “adequate time to address lifestyle issues with patients” as a perceived barrier to managing patients with CVD.^27^ Clinicians' insufficient training and lack of self‐confidence in preventive behavioral counseling have been identified as an additional barrier to the delivery of preventive services by the US Preventive Services Task Force.^28^ The AHA and health systems should consider these practical issues as they work toward having the cardiovascular health metrics used widely as a tool of population‐level cardiovascular health assessment and improvement.

### Limitations

First, the information collected from the HONU analysis and the levels of adherence to the AHA's 7 metrics of ideal cardiovascular health have limited generalizability to other population samples within the United States due to the racial homogeneity of the New Ulm community. However, the number of ideal cardiovascular health metrics met by the HONU population could be a benchmark of what levels of cardiovascular health might be expected from other rural, predominantly white communities. Next, the data collected at community health screenings tend to carry a healthy participant bias,^29–30^ especially compared with the data collected from the NHANES, which analyzed data from a nationally representative sample of individuals. As evidence of the healthy participant effect, the proportion of current smokers among the HONU sample was only 10.0%, whereas prevalence of smokers in the general Minnesota population is 16.1%.^31^ On a related note, given that individuals included in the HONU analysis comprise only 36% of the total New Ulm, Minn, population, the data collected may not be representative of the cardiovascular health of the New Ulm population. Fourth, our study used a more limited nutrition measure than that of the AHA, using fruit and vegetable intake as a proxy for overall healthy diet score, rather than including the 5 elements of the DASH diet that include fruit and vegetable intake, whole grain consumption, fish consumption, limited sodium intake, and limited sugar‐sweetened beverage intake.^32–33^ However, the authors thought that the existing literature has established the relationship between high fruit and vegetable intake with reduced risk of CHD, which justified the substitution.^34–35^ Finally, the data collected on behavioral health measures depended on accurate self‐reporting by the screening participants. However, this limitation exists for all studies that use the AHA's cardiovascular health metrics and is a limitation of the tool as a whole. Despite these limitations, the findings of the study remain an important contribution to the existing literature and demonstrate how these measures can be used in a community‐based CHD prevention program.

## Conclusion

Our study indicated that baseline levels of ideal cardiovascular health in a rural population are low and approximately equivalent to those found in the American population at large, although more favorable than those found in an urban American community. Additionally, the study demonstrated the utility of completing an analysis of the population based on the AHA's 7 cardiovascular health metrics for both research and practical purposes, to evaluate the baseline cardiovascular health of the population and help inform development of community interventions to decrease the cardiovascular disease risk factors most prevalent in the community. To achieve the AHA 2020 Impact Goals, it will be important to continue to improve the health of individuals through policy, systems, and environmental approaches that make it easier for providers to address risks with patients and easier for patients to make healthier lifestyle choices.
